# Fat Matters: Exploring Cancer Risk through the Lens of Computed Tomography and Visceral Adiposity

**DOI:** 10.3390/jcm13020453

**Published:** 2024-01-14

**Authors:** Federico Greco, Claudia Lucia Piccolo, Valerio D’Andrea, Arnaldo Scardapane, Bruno Beomonte Zobel, Carlo Augusto Mallio

**Affiliations:** 1 Department of Radiology, Cittadella della Salute Azienda Sanitaria Locale di Lecce, Piazza Filippo Bottazzi 2, 73100 Lecce, Italy; 2 Research Unit of Radiology, Department of Medicine and Surgery, Università Campus Bio-Medico di Roma, Via Alvaro del Portillo 21, 00128 Roma, Italy; c.piccolo@policlinicocampus.it (C.L.P.); b.zobel@policlinicocampus.it (B.B.Z.); c.mallio@policlinicocampus.it (C.A.M.); 3 Fondazione Policlinico Universitario Campus Bio-Medico, Via Alvaro del Portillo 200, 00128 Roma, Italy; 4 Dipartimento Interdisciplinare di Medicina, Sezione di Diagnostica per Immagini, Università degli Studi di Bari “Aldo Moro”, Piazza Giulio Cesare 11, 70124 Bari, Italy; arnaldo.scardapane@gmail.com

**Keywords:** adipose tissue, body composition, cancer, computed tomography, fat, obesity paradox, oncologic disease, precision medicine, visceral adipose tissue, visceral obesity

## Abstract

Obesity is an established risk factor for cancer. However, conventional measures like body mass index lack precision in assessing specific tissue quantities, particularly of the two primary abdominal fat compartments, visceral adipose tissue (VAT) and subcutaneous adipose tissue (SAT). Computed tomography (CT) stands as the gold standard for precisely quantifying diverse tissue types. VAT, distinguished by heightened hormonal and metabolic activity, plays a pivotal role in obesity-related tumor development. Excessive VAT is linked to aberrant secretion of adipokines, proinflammatory cytokines, and growth factors, fostering the carcinogenesis of obesity-related tumors. Accurate quantification of abdominal fat compartments is crucial for understanding VAT as an oncological risk factor. The purpose of the present research is to elucidate the role of CT, performed for staging purposes, in assessing VAT (quantity and distribution) as a critical factor in the oncogenesis of obesity-related tumors. In the field of precision medicine, this work takes on considerable importance, as quantifying VAT in oncological patients becomes fundamental in understanding the influence of VAT on cancer development–the potential “phenotypic expression” of excessive VAT accumulation. Previous studies analyzed in this research showed that VAT is a risk factor for clear cell renal cell carcinoma, non-clear cell renal cell carcinoma, prostate cancer, and hepatocarcinoma recurrence. Further studies will need to quantify VAT in other oncological diseases with specific mutations or gene expressions, in order to investigate the relationship of VAT with tumor genomics.

## 1. Introduction

Energy intake greater than energy consumption, resulting from the balance between physical and metabolic activity, can result in obesity. This causes excessive or anomalous accumulation of adipose tissue that exceeds genetically and epigenetically determined fat deposits. This accumulation of fat could lead to increased risk for many pathologies [1]. Obesity is extremely widespread globally and is a serious public health problem. In addition to being a known risk factor for various chronic diseases such as cardiovascular diseases and type 2 diabetes, obesity has been recognized as a risk factor for cancer [1,2]. A body mass index (BMI) greater than 30 kg/m^2^ defines obesity, while being overweight is characterized by having a BMI higher than 25 kg/m^2^ [3]. However, BMI does not provide quantitative data for the distribution of different tissues, and nor does it allow us to distinguish the two main compartments of abdominal adipose tissue, visceral adipose tissue (VAT) and subcutaneous adipose tissue (SAT), separately [4]. This distinction is of considerable importance, especially in light of several recent studies that have highlighted the importance of VAT, the fat compartment located around internal abdominal organs, in carcinogenesis. VAT and SAT have different characteristics; in particular, VAT has molecular, cellular, and anatomical characteristics denoting greater hormonal and metabolic activity compared to SAT [5,6]. Moreover, BMI is liable to underestimate the prevalence of visceral obesity in the population through misclassification, leading to potential bias regarding the association between obesity and cancer towards the null effect [7]. Excessive amounts of VAT results in the secretion of adipokines, proinflammatory cytokines, and growth factors, known mediating molecules impacting the oncogenesis of obesity-related tumors [8,9]. Computed tomography (CT) and magnetic resonance imaging (MRI) are gold standard imaging techniques for non-invasive tissue quantification [10,11,12]. The quantification of VAT using the CT approach provides data on the phenotypic expression of pathological processes that underlie the development of obesity-related tumors. The aim of this literature review is to explore the role of VAT in oncologic diseases through examining how visceral adiposity impacts cancer risk, elucidating the mechanisms underlying such association with cancer, and interpreting the clinical implications for cancer prevention and treatment. The motivation of this literature review is to bridge the gap in understanding the role of VAT across different cancer types and offer a comprehensive view on the topic. This review incorporates a broad range of studies and findings, from epidemiological observations to molecular and cellular mechanisms, to provide an overview of the subject.

## 2. Methods

This literature review was performed in November 2023 using MEDLINE PubMed Central and Google Scholar, taking into account only articles written in English and without limits of time span. Keywords used for the search included “adipose tissue”, “visceral adiposity”, “visceral adipose tissue”, “intra-abdominal fat”, “cancer”, “oncologic disease”, “obesity”, and “cancer risk”. Relevant articles related to visceral adipose tissue and cancer were selected and included. This is a narrative review; articles selected for inclusion in this study were agreed upon by the authors (F.G. and C.A.M.) who conducted the initial search. Disagreements about inclusion and exclusion of articles were resolved through a Delphi consensus achieved with all other authors. Reporting of the results is in alignment with the SANRA checklist for narrative review [13].

## 3. Visceral Adiposity and Cancer Development

Lu et al. performed a Mendelian randomization study to evaluate causal effects of visceral adipose tissue on risk of cancers [14]. Two-sample Mendelian randomization analyses were conducted to assess the causal effects of VAT on six common cancers (i.e., lung, breast, pancreatic, colorectal, ovarian, and prostate cancers). Genetic association data relating to these six tumor types were obtained from large-scale consortia with an average of 19,576 cases and 43,272 controls [14]. The researchers utilized five Mendelian randomization methods [inverse-variance weighted, Mendelian randomization-Egger regression, weighted median, Mendelian randomization-Pleiotropy Residual Sum and Outlier (Mendelian randomization-PRESSO) and Radial mendelian randomization] [14]. To estimate the effect of VAT independently of BMI, multivariable Mendelian randomization was used [14]. A series of sensitivity analyzes were consequently performed as validation for the primary Mendelian randomization results [14]. In the false discovery rate correction for multiple testing (*q*-value < 0.05), two associations survived: using the inverse-variance weighted method, the odds ratios (95% CIs) per unit increase in genetically determined VAT were 1.65 (1.03 to 2.62) for pancreatic cancer and 1.47 (1.20 to 1.82) for lung squamous-cell carcinoma, respectively, with the same directions and overlapped confidence intervals as the Mendelian randomization-Egger regression and weighted median results [14]. No evidence was found for the causal effect of VAT on the risk of other types of cancers [14].

### 3.1. Visceral Adipose Tissue and Lung Cancer Risk

Lung squamous-cell carcinoma originates from squamous metaplasia of the bronchial epithelium, which presents a greater vulnerability to environmental factors [15,16,17]. Cytokines and adipokines secreted by VAT promote DNA damage and dysregulate DNA repair mechanisms, increasing mutation rates that lead to carcinogenesis [18]. Mallio et al. evaluated the distribution of abdominal adipose tissue in patients with non-small cell lung cancer (NSCLC). This study found no significant difference in VAT, SAT, and total adipose tissue (TAT) between NSCLC patients and control groups [19]. The proportion of VAT observed was similar between patients (considering male and female patients together) and controls; furthermore, the controls showed a greater quantity of SAT and consequently of TAT [19]. These differences, although not statistically significant, suggest a preservation of VAT in NSCLC patients with respect to the amount of SAT and TAT [19]. Considering male and female NSCLC patients separately, female NSCLC patients showed lower mean VAT values than the control group; on the other hand, SAT values were higher in patients than controls. Male NSCLC patients showed lower SAT and slightly higher VAT values compared to the control group [19]. Moreover, considering male and female together, a slightly higher VAT:SAT ratio in NSCLC patients than the control group was detected [19]. A higher VAT:SAT ratio was found in the female control group compared to the patients, while in the male group a higher VAT:SAT ratio was found in the patients compared to the control group [19]. The analysis of this study highlighted that VAT cannot be considered a risk factor for NSCLC development. The results of this study are summarized in Table 1.

### 3.2. Visceral Adipose Tissue and Breast Cancer Risk

Lu et al. found no causal effect of VAT on breast cancer development [14], although other studies found a correlation [20,21]. The increase in size of adipocytes induces hypoxia in the surrounding microenvironment, which determines the greater expression of hypoxia-inducible factor-1 alpha (HIF-α) in adipocytes and in turn upregulates the expression of proinflammatory factors tumor necrosis factor-alpha (TNF-α), interleukin-6 (IL-6), and monocyte chemo-attracting protein-1 (MCP-1). The development of a chronic inflammatory state can determine breast cancer development, progression, treatment resistance, and metastasis [22]. A case-control study conducted covering 234 newly diagnosed breast cancer patients and 211 controls in the Republic of Korea evaluated abdominal adipose tissue distribution using CT scans. No statistically significant correlation was found between the quantity of VAT and breast cancer risk. It was found an increased VAT in postmenopausal women by 1.50 (95% CI, 0.75–2.98), while in premenopausal women this effect was not found [23]. Furthermore, a high histological grade score (≥2) was associated with high VAT and a slightly increased mortality rate (r = 0.15, *p* = 0.07) [24]. Patients with negative progesterone receptors tended to have a higher fat ratio [Fat ratio% = (VAT/TAT) × 100] than patients with positive progesterone receptors [23]. Especially in premenopausal patients, higher distribution of abdominal fat ratio had a stronger effect on negative than positive HRs [estrogen receptor (ER), *p* = 0.12; progesterone receptor (PR), *p* = 0.06; human epidermal growth factor receptor 2 (HER2), *p* = 0.43] [23]. In this study, VAT was not shown to be a risk factor for the development of breast cancer. The results of this study are summarized in Table 2.

### 3.3. Visceral Adipose Tissue and Colorectal Cancer Risk

**Figure 1:**
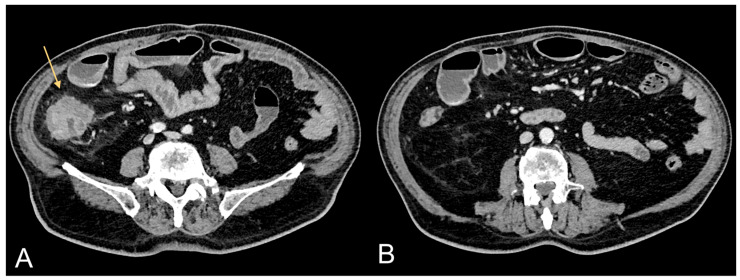
Contrast-enhanced CT images of a 75-year-old male patient showing CRC (light orange arrow in (**A**)) and associated excessive amount of VAT (**B**).

No association was found between VAT and the risk of colorectal cancer [14]. However, it has been suggested that the amount of VAT and the VAT:SAT index could be correlated with the pathogenesis of colorectal cancer more so than BMI [24]. VAT, given its adjacency to the abdominal organs and tissues, could interact with them. For example, the proximity of VAT to the portal vein could be responsible for the fact that fatty acids and inflammatory factors, such as cytokines, are conveyed into the liver, possibly leading to hepatic misfunction and injury. This delivery of lipids to the tissues by excess VAT creates a state of low-grade inflammation, favorable to tumor development [25,26]. Furthermore, the switch of M2 macrophages (anti-inflammatory and adipostatic) to the M1 phenotype of macrophages (proinflammatory and proadipogenic) infiltrating the VAT creates an inflammatory microenvironment that determines an increased release of cytokines and other inflammatory factors that promote tumor growth [25]. “Adipose tissue remodeling” occurs when there is an excess of fat: an increase in the number and size of mature adipocytes occurs when cells of the stromal vascular fraction, which contains heterogeneous cell populations, including preadipocytes, endothelial cells, pericytes, and immune cells (macrophages, T-cells, neutrophils, and lymphocytes) are recruited, and consequently adipocytes secrete a series of adipokines that differentiate adipocytes into mature adipocytes [24,27]. Secreted adipokines and cytokines contribute to low-grade systemic inflammation associated with visceral obesity, relevant to the development of colorectal cancer (Figure 1) [25]. Adipokines and cytokines are also overproduced in states of hypoxia and oxidative stress due to inadequate vascularization caused by the increase in adipose tissue [25]. Overall, all of these factors can determine hyperplasia, proliferation, and carcinogenesis in colon cells [25]. Erarslan et al. evaluated the quantity of VAT in colorectal adenoma patients, colorectal carcinoma patients, and control groups, finding no statistically significant correlation [28]. In this study, no statistically significant correlation was demonstrated between the amount of VAT and the development of colorectal cancer. The results of this study are summarized in Table 3.

### 3.4. Visceral Adipose Tissue and Pancreatic Cancer Risk

Since VAT and pancreatic tissue are adjacent, they can interact with each other. Increased VAT results in fatty infiltration of the pancreas and is correlated with pancreatic intraepithelial neoplasia that has a high risk of transformation into pancreatic ductal adenocarcinoma [29]. A systematic review that evaluated the relationship between intrapancreatic adipose tissue and pancreatic cancer or premalignant lesions of the pancreas found 13 studies that showed the prevalence of intra-pancreatic adipose tissue deposition in patients with pancreatic cancer or pre-malignant lesions to be 52% (95% confidence interval, 38–66%). Pancreatic cancer or pre-malignant lesions was related with a significantly increased risk of intra-pancreatic adipose tissue deposition [relative risk 2.78 (95% confidence interval, 1.56–4.94, *p* < 0.001)] [30]. This suggests that the local environment contributes to tumor development and progression. It is likely that the mechanism through which intra-pancreatic adipose tissue contributes to the carcinogenesis of pancreatic cancer is not dissimilar to any other adipose tissue in relation to obesity-associated cancers and is based mainly on fat inflammation, in which there is an increased release of cytokines such as tumor necrosis factor-α, C-C motif chemokine ligand 2, and leptin, which determine a pro-inflammatory microenvironment that contributes to carcinogenesis [30,31,32,33]. Greco et al. quantified abdominal TAT, VAT, and SAT and calculated VAT:SAT ratio in 20 male pancreatic cancer patients and in 20 male patients from a control group. The results did not show a statistically significant difference regarding the quantity of VAT and the VAT:SAT ratio, while a statistically significant decrease in SAT was found in the pancreatic cancer patients; TAT was slightly above the threshold of significance in these patients due to the SAT reduction [34]. This study showed no statistically significant correlation between the amount of VAT and the development of pancreatic cancer. The results of this study are summarized in Table 4.

### 3.5. Visceral Adipose Tissue and Liver Cancer Risk

**Figure 2:**
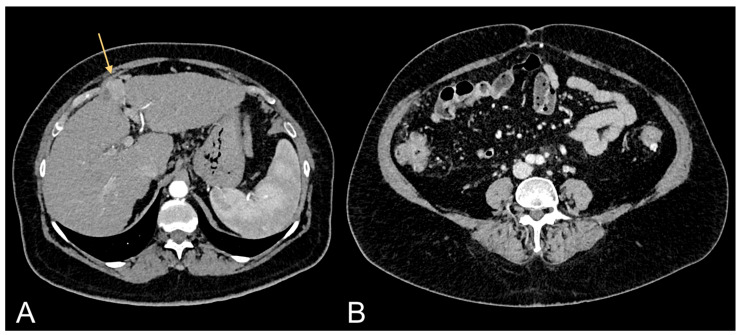
Contrast-enhanced CT images of a 69-year-old female patient showing HCC in cirrhotic liver (light orange arrow in (**A**)) and relative excessive amount of VAT (**B**).

It is known that a high amount of VAT is causally associated with a higher risk of non-alcoholic fatty liver disease (NAFLD) [35]. NAFLD is a clinical condition that can progress to non-alcoholic steatohepatitis (NASH), another important liver disease that precedes cirrhosis and hepatocellular carcinoma (HCC) [36]. Severity of NAFLD in obese and non-obese patients without viral hepatitis is positively related to visceral fat accumulation and insulin resistance [37]. This suggests that hepatic fat infiltration in NAFLD may depend on visceral fat accumulation independently of BMI [38]. Ohki et al. quantified VAT in patients undergoing percutaneous radiofrequency ablation for HCC to assess whether visceral fat accumulation increases the risk of recurrence in HCC patients with suspected NASH [38]. The patients were divided into two groups: a high VAT area group, defined as VAT area > 30 cm^2^ in males and VAT area > 90 cm^2^ in females (n = 27), and the control group (n = 35) [38]. A statistically significant difference for HCC recurrence risk was found between the two groups: risk rates of 15.9%, 56.5%, and 75.1% at 1, 2 and 3 years, respectively, in the high VAT area group, and 9.7%, 31.1%, and 43.1%, respectively, in the control group (*p* = 0.018) [38]. This demonstrates that VAT is an independent risk factor of HCC recurrence after treatment in patients with suspected NASH [38]. It may be suspected that high amounts of VAT affected the effectiveness of percutaneous radiofrequency ablation; however, only two cases of local tumor progression were found. Patients with higher amounts of VAT showed a higher incidence of intrahepatic recurrence distant from the primary lesion, suggesting that increased VAT affects metachronous de novo carcinogenesis [38]. This study showed a statistically significant correlation between the amount of VAT and the development of HCC recurrence. The results of this study are summarized in Table 5. Further studies should quantify VAT in viral hepatitis patients (e.g., post-infection with hepatitis virus B or C) with HCC, and in alcoholic steatohepatitis patients with HCC, versus those that develop cancer without those predisposing factors (Figure 2).

### 3.6. Visceral Adipose Tissue and Kidney Cancer Risk

The role of VAT in the pathogenesis of clear cell renal cell carcinoma (ccRCC) is well known. Greco et al. quantified abdominal adipose tissue compartments (VAT, SAT, and TAT areas) in ccRCC patients using a CT approach [39]. Two groups were included in the study, one consisting of 10 ccRCC patients, and the other of 35 controls. Statistically significant differences between ccRCC patients and controls were obtained for VAT area (*p* = 0.01) and VAT:SAT ratio (*p* < 0.05). No significant difference was obtained for TAT and SAT areas [39]. To confirm and support these results, another study quantified VAT, SAT, and TAT in ccRCC patients (n = 106) and a control group (n = 35). The ccRCC group was further divided into two subgroups: ccRCC patients without peritumoral collateral vessels (n = 48) and ccRCC patients with peritumoral collateral vessels (n = 58) [40]. Collateral vessels are enlarged renal capsular veins macroscopically visible in CT or MRI studies, which are correlated with high Fuhrman grade (e.g., III and IV) in ccRCC [41]. The study showed statistically significant differences between the ccRCC group and control group for TAT area (*p* < 0.005), VAT area (*p* < 0.005) and SAT area (*p* = 0.01), between ccRCC patients without peritumoral collateral vessels and control group for TAT area (*p* < 0.001), VAT area (*p* = 0.005), and SAT area (*p* = 0.001), and between ccRCC patients with peritumoral collateral vessels and the control group for TAT area (*p* = 0.01) and VAT area (*p* = 0.01) [37]. Furthermore, ccRCC patients with peritumoral collateral vessels present a lower amount of SAT and abdominal muscle mass compared to ccRCC patients without peritumoral collateral vessels [12,42]. Greco et al. also quantified abdominal adipose tissue compartments in non-ccRCC (nccRCC) patients, detecting statistically significant differences in TAT area, VAT area, and VAT:SAT ratio in nccRCC patients compared to the control group (*p* = 0.059, *p* = 0.008, and *p* = 0.022), due to a greater amount of VAT in nccRCC patients [11]. These studies demonstrated statistically significant correlations between the amount of VAT and the development of ccRCC and non-ccRCC. The results of this study are summarized in Table 6.

### 3.7. Visceral Adipose Tissue and Ovarian Cancer Risk

Lu et al. found no causal effects between VAT and ovarian cancer risk [14]. No articles were found regarding the quantification of VAT in patients with a first diagnosis of ovarian cancer in order to evaluate the possible action of visceral adiposity in carcinogenesis. The role of obesity in ovarian cancer risk is controversial [43]. A systematic review analyzed 43 studies that included body mass index as a measure of exposure and the majority were based on self-reported measures by participants [43]. This review concluded that there is limited and inconsistent evidence on obesity and the risk of ovarian cancer [43]. Quantification of abdominal adipose tissue compartments should be evaluated to verify the possible influence of VAT on the risk of developing ovarian cancer.

### 3.8. Visceral Adipose Tissue and Prostate Cancer Risk

Lu et al. found no statistically significant correlation between VAT and prostate cancer risk [14]. This type of association was also studied by von Hafe et al. using CT [44]. In particular, quantitative analysis of the abdominal adipose tissue compartments was carried out in 63 patients with prostate cancer and 63 control subjects [44]. Prostate cancer patients showed a significantly higher mean TAT area (509.2 ± 226.1 vs. 334.3 ± 132.9 cm^2^, *p* < 0.001), mainly due to higher mean VAT area values (324.7 ± 145.6 vs. 177.4 ± 88.4 cm^2^, *p* < 0.001). The mean observed VAT:SAT ratio was also higher in prostate cancer patients (1.8 ± 0.4 vs. 1.2 ± 0.4, *p* < 0.001). Participants with high values of VAT area and VAT:SAT ratio presented a significantly increased risk of prostate cancer (odds ratio 4.6; 95% confidence interval 2.6 to 8.2 per SD increase for VAT area and odds ratio 6.0; 95% confidence interval 2.3 to 11.0 for SD increase for VAT:SAT ratio) [44]. These results suggest the role of VAT as a risk factor for prostate cancer [44]. This study showed a statistically significant correlation between the amount of VAT and the development of prostate cancer. The results of this study are summarized in Table 7.

## 4. Visceral Adiposity and Cancer Survival

The most comprehensive study analyzing the quantification of adipose tissue in relation to cancer-related outcomes showed that VAT, SAT, and TAT had no significant correlation with cancer progression, death from primary cancer, or mortality. The tumors analyzed were head and neck cancer, breast cancer, gastroesophageal cancer, lung cancer, hepatocarcinoma, pancreatic cancer, renal cancer, and prostate cancer [45]. SAT appeared protective in head and neck, gastroesophageal, ovarian, and prostate cancer, being correlated with significantly lower mortality risk [45]. High amounts of adipose tissue could be associated with greater muscle mass that develops to support the increased weight. Often, decreased muscle mass determines a greater risk of recurrence and a higher degree of overall and cancer-specific mortality [46]. Therefore, unless there is a severe increase in adipose tissue, excess fat could be attenuated by an increase in muscle mass which has been shown to be protective [47]. Furthermore, an increase in adiposity, in particular in SAT, can provide protective nutritional reserves, as this compartment type is a more inert storage depot than VAT and is not so powerfully linked to adverse metabolic effects [48]. In addition, tumor biology may be related to excess adipose tissue resulting in heterogeneity in disease [49]. For example, clear cell renal cell carcinoma (ccRCC) patients with von Hippel-Lindau (VHL) gene mutation showed a better survival trend compared with ccRCC patients with titin (TTN) gene mutation. Not surprisingly, ccRCC patients with VHL gene mutation presented higher VAT, SAT, TAT, and muscle mass than ccRCC patients with TTN gene mutation [50]. This introduces the concept of obesity paradox in which tumors respond better in obese patients. The average reference point for normal weight is considered to be a BMI of 22.5 kg/m^2^ [51]. When patients with BMI values greater than 22.5 kg/m^2^ present a significant reduction in the risk of mortality, the obesity paradox occurs [52].

**Figure 3:**
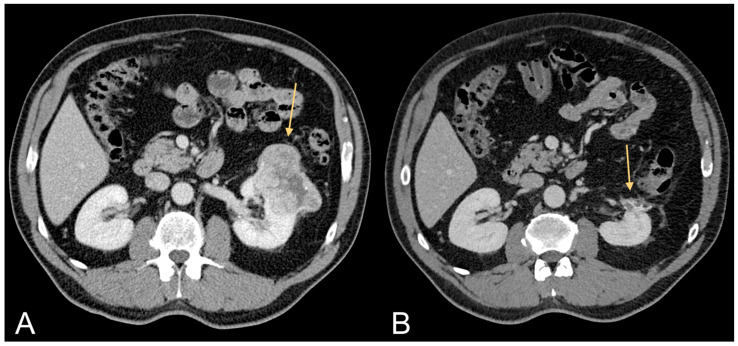
Contrast-enhanced CT images of a 63-year-old male patient with excessive amount of VAT showing ccRCC (light orange arrow in (**A**)) and follow-up after tumorectomy (light orange arrow in (**B**)).

For example, it has been shown that obese RCC patients respond better to treatment than normal-weight RCC patients in that they present better overall survival (OS) and cancer-specific survival (CSS) rates (Figure 3) [53]. Kamat et al. showed a protective effect of adiposity in RCC patients with evidence of longer overall survival in overweight and obese RCC patients compared to normal weight RCC patients [54]. A multivariable analysis performed on 1543 nephrectomized RCC patients showed significantly longer OS and CSS in obese RCC patients than normal weight RCC patients [55]. Tsang et al. performed a retrospective study to evaluate the association between BMI and OS in patients with distant metastases. Underweight patients showed a median OS time of 3.23 months (range: 0.1–122.17), normal weight patients a median OS time of 6.08 months (range: 0.03–149.46), overweight patients a median OS time of 7.99 months (range: 0.07–158.01), and obese patients a median OS time of 12.49 months (range, 0.2–164.1)(log-rank: *p* < 0.001). Obese and overweight patients showed a reduced risk of all-cause mortality in multivariable analysis compared to normal-weight patients (HR = 0.676; 95% *p* < 0.001 and HR = 0.84; *p* < 0.001, respectively). Underweight patients showed a significantly higher risk for all causes of death (HR = 1.41; *p* < 0.001) [55]. A systematic review and meta-analysis performed on the obesity paradox in CRC patients showed that a high BMI significantly associated with more favorable outcomes in CRC patients [26]. Sixteen studies were included (with a total of 55,391 patients). Underweight patients showed worse OS (HR¼1.26; 95%CI, 1.15–1.37) and disease-free survival (DFS) (HR¼1.19; 95%CI, 1.11–1.27) compared to normal weight patients [56]. Overweight patients showed better OS (HR¼0.92; 95%CI, 0.86–0.99), DSF (HR¼0.96; 95%CI, 0.93–1.00), and CSS (HR¼0.86; 95%CI, 0.76–0.98) [57]. Compared to normal weight patients, patients with morbid obesity had worse OS (HR¼1.12; 95%CI, 1.02–1.22) and DFS (HR¼1.15; 95%CI, 1.07–1.24) [56]. Although obesity is a determining risk factor for the development of CRC cancer, high BMI presents reduced mortality compared to normal weight patients [56].

## 5. Unmet Needs and Future Developments

The definition of VAT measured from CT depends upon the Hounsfield Unit (HU) range and the axial level of reference (e.g., lower thoracic versus lumbar vertebrae) used to define and extract fat pixels from imaging, with the extreme values of this range being −250HU to −30HU and T10 to L4/L5 levels, depending on the studies [57]. Hence, there is interest in considering the impact of contrast media on the ability of CT and MRI to detect VAT and SAT. The literature indicates that VAT and SAT are higher in non-contrast scans compared to studies reporting on measurements taken from contrast sequences, whereas the radiation attenuation is lower in contrast scans compared to plain CT: in fact, the impact of contrast media is proportional to the density of VAT and SAT because fat with higher density tends to show greater measurement differences between scans with and without contrast [57]. Therefore, to assess the correlation between VAT and SAT and cancer, one should standardize measurements for patients with low and high density fat, keeping into account the measurement variability that axial levels of reference and contrast versus non-contrast scans can give rise to. Additionally, MRI sequences can also provide accurate means for determining absolute fat mass in organs, muscles, and adipose and lean tissues. For instance, proton density fat fraction can indicate the relative fat content on a voxel-by-voxel basis [58]. In real life, dynamic contrast enhanced (DCE) MRI examinations are the most commonly performed for oncological purposes, usually with the inclusion of fat suppression sequences; this option provides better time-signal intensity curves and allows radiologists to qualitatively assess the kinetics of the contrast media uptake in tumors. On one hand, DCE-MRI and fat fraction imaging are known to provide useful information about vascular perfusion kinetics in brown and beige adipose tissues [59]; on the other hand, the use of nano-coating to enhance the performance of contrast media will open new horizons in the diagnosis of patients with cancer [60]. Given all the above, future research will necessarily have to explore the impact of contrast enhanced CT and MRI, and the role that optimized contrast media might have in obtaining better insights into the metabolic relevance of VAT/SAT in oncological processes.

## 6. Conclusions

In conclusion, this analysis underscores the significant impact of visceral adipose tissue on the development of obesity-related tumors, shedding light on its intricate association with genetic and environmental factors and patient prognosis. Addressing the identified flaws in current studies enhances our understanding, emphasizing the necessity for precise quantification in various clinical scenarios for improved risk assessment and personalized treatment strategies.

## Figures and Tables

**Table 1:** Summary of studies evaluating quantity of VAT in NSCLC patients.

Authors	Cancer	Imaging Technique	Level	Number and Gender of Patients and Controls	Results
Mallio et al. (2018) [19]	NSCLC female patients - 33 adenocarcinomas - 8 squamous cell carcinomas - 4 NSCLCs NSCLC male patients - 19 adenocarcinomas - 1 squamous cell carcinomas - 1 NSCLCs	CT	L3	42 male patients of control group 28 female patients of control group 45 male NSCLC patients 21 NSCLC female patients	No significative statistical difference between patients (male and female) and control group (male and female) (*p* = 0.99). No significative statistical difference between male patients and male control group (*p* = 0.88). No significative statistical difference between female patients and female control group (*p* = 0.43).

Abbreviations: CT, computed tomography; NSCLC, non-small cell lung cancer; VAT, visceral adipose tissue.

**Table 2:** Summary of studies evaluating quantity of VAT in breast cancer patients.

Authors	Cancer	Imaging Technique	Level	Number and Gender of Patients and Controls	Results
Kim et al. (2019) [23]	234 breast cancer patients	CT	Umbilicus or L4	234 breast cancer patients 211 control group	No significative statistical difference between total patients and total control group (*p* = 0.64). No significative statistical difference between premenopausal patients and premenopausal control group (*p* = 0.34). No significative statistical difference between postmenopausal patients and postmenopausal control group (*p* = 0.34).

Abbreviations: CT, computed tomography; VAT, visceral adipose tissue.

**Table 3:** Summary of studies evaluating quantity of VAT in colorectal cancer patients.

Authors	Cancer	Imaging Technique	Level	Number and Gender of Patients and Controls	Results
Erarslan et al. (2009) [28]	31 colorectal adenoma patients 23 colorectal carcinoma patients	CT	L4	27 male patients of control group 23 female patients of control group 17 colorectal adenoma male patients 14 colorectal adenoma female patients 14 colorectal carcinoma male patients 9 colorectal carcinoma female patients	No significative statistical difference between colorectal adenoma patients and control group (*p* = 0.64). No significative statistical difference between colorectal carcinoma patients and control group (*p* = 0.15).

Abbreviations: CT, computed tomography; VAT, visceral adipose tissue.

**Table 4:** Summary of studies evaluating quantity of VAT in pancreatic cancer patients.

Authors	Cancer	Imaging Technique	Level	Number and Gender of Patients and Controls	Results
Greco et al. (2019) [11]	20 pancreatic adenocarcinoma patients	CT	3 cm above the lower margin of L3	20 male patients of control group 20 pancreatic adenocarcinoma male patients	No significative statistical difference between pancreatic adenocarcinoma patients and control group (*p* = 0.23).

Abbreviations: CT, computed tomography; VAT, visceral adipose tissue.

**Table 5:** Summary of studies evaluating quantity of VAT in HCC patients.

Authors	Cancer	Imaging Technique	Level	Number and Gender of Patients and Controls	Results
Ohki et al. (2009) [37]	62 HCC patients	CT	Umbilicus	40 male HCC patients 22 female HCC patients	VAT showed a significative statistical correlation as a risk factor of HCC recurrence (risk ratio 1.08, per 10 cm^2^, *p* = 0.046).

Abbreviations: CT, computed tomography; HCC, hepatocellular carcinoma; VAT, visceral adipose tissue.

**Table 6:** Summary of studies evaluating quantity of VAT in kidney cancer patients.

Authors	Cancer	Imaging Technique	Level	Number and Gender of Patients and Controls	Results
Greco et al. (2018) [38]	10 ccRCC patients	CT	3 cm above the lower margin of L3	35 male patients of control group 10 male ccRCC patients	Significative statistical difference between ccRCC patients and control group (*p* = 0.015).
Greco et al. (2018) [39]	106 ccRCC patients	CT	3 cm above the lower margin of L3	35 male patients of control group 106 male ccRCC patients	Significative statistical difference between ccRCC patients and control group (*p* = 0.004).
Greco et al. (2020) [40]	13 pRCC patients 13 chRCC patients	CT	3 cm above the lower margin of L3	35 male patients of control group 26 male nccRCC patients	Significative statistical difference between nccRCC patients and control group (*p* = 0.008).

Abbreviations: ccRCC, clear cell renal cell carcinoma; chRCC, chromophobe renal cell carcinoma; CT, computed tomography; pRCC, papillary renal cell carcinoma; VAT, visceral adipose tissue.

**Table 7:** Summary of studies evaluating quantity of VAT in prostate cancer patients.

Authors	Cancer	Imaging Technique	Level	Number and Gender of Patients and Controls	Results
von Hafe et al. (2004) [43]	63 prostate cancer patients	CT	L4	63 patients of control group 63 prostate cancer patients	Significative statistical difference between prostate cancer patients and control group (*p* < 0.001).

Abbreviations: CT, computed tomography; VAT, visceral adipose tissue.
